# Depression, diabetes and change in cognitive functioning: results from the Canadian Longitudinal study on Aging

**DOI:** 10.1093/eurpub/ckac129.702

**Published:** 2022-10-25

**Authors:** M Shojaa, S Deschenes, J McLeish, N Schmitz

**Affiliations:** 1 Tuebingen University, Population-Based Medicine, Tuebingen, Germany; 2 McGill University, Psychiatry & Epidemiology, Montreal, Canada; 3 University College Dublin, Psychology, Dublin, Ireland

## Abstract

**Background:**

Individuals who ultimately receive a diagnosis of dementia typically have an observable accelerated cognitive decline (ACD) many years prior to diagnosis. Depression in combination with diabetes is an emerging risk factor that is associated with cognitive problems. Using data from the Canadian Longitudinal Study on Aging, the objective of the present study was to investigate the longitudinal association between depression, diabetes, and cognitive decline in an elderly cohort.

**Methods:**

Baseline and follow-up data from a population-based study in Canada were used. The sample consisted of 18161 adults between 45 and 85 years of age without diabetes. Cognitive functioning was assessed at baseline and after 4 years using six measures: the Rey Auditory Verbal Learning Test (RAVLT), the Mental Alternation Test (MAT), the Animal Fluency Test (AF), the Controlled Oral Word Association Test (COWAT), the Stroop Test, and the Prospective Memory Test. Depression was assessed using the CES-D10. Regression analysis was conducted to evaluate interactions between depression, diabetes and cognitive decline.

**Results:**

The mean age of participants was 61 years. Participants with a comorbidity of depression and diabetes had an accelerated cognitive decline (g-factor) compared to those with depression without diabetes and those with diabetes without depression (regression coefficients ß=-0.145 (0.036), ß=-0.076 (0.011), and ß=-0.053 (0.021), respectively).

**Conclusions:**

This study suggests that depression and diabetes might increase the risk of cognitive decline in a synergistic way.

